# Immuno-epidemiological Modeling of HIV-1 Predicts High Heritability of the Set-Point Virus Load, while Selection for CTL Escape Dominates Virulence Evolution

**DOI:** 10.1371/journal.pcbi.1003899

**Published:** 2014-12-18

**Authors:** Christiaan H. van Dorp, Michiel van Boven, Rob J. de Boer

**Affiliations:** 1 Theoretical Biology and Bioinformatics, Universiteit Utrecht, Utrecht, The Netherlands; 2 National Institute for Public Health and the Environment, Bilthoven, The Netherlands; ETH Zurich, Switzerland

## Abstract

It has been suggested that HIV-1 has evolved its set-point virus load to be optimized for transmission. Previous epidemiological models and studies into the heritability of set-point virus load confirm that this mode of adaptation within the human population is feasible. However, during the many cycles of replication between infection of a host and transmission to the next host, HIV-1 is under selection for escape from immune responses, and not transmission. Here we investigate with computational and mathematical models how these two levels of selection, within-host and between-host, are intertwined. We find that when the rate of immune escape is comparable to what has been observed in patients, immune selection within hosts is dominant over selection for transmission. Surprisingly, we do find high values for set-point virus load heritability, and argue that high heritability estimates can be caused by the ‘footprints’ left by differing hosts' immune systems on the virus.

## Author Summary

HIV-1 is a relatively young virus, being introduced in the human population somewhere between 1884 and 1924. Yet, previous studies suggest that the virus has already evolved to be efficiently transmitted among humans. Efficient transmission occurs when the set-point virus load, the semi-stable number of virus particles in the blood during the asymptomatic phase, is intermediate (approximately  particles/ml). At this virus load level, individuals remain asymptomatic for a long period (7.0 years on average), and still remain sufficiently infectious. In this study, we model the combined immunological and epidemiological dynamics of HIV-1 to explore whether population-level adaptation is feasible. We show that strong selective forces within the host are expected to dominate the much weaker population-level selection, unless the within-host dynamics of immune escape becomes exceedingly slow. Surprisingly, our analyses yield high levels of set-point virus load heritability, as observed in human populations. In the model, heritability of set-point virus load partially results from an immunological ‘footprint’ of the host-virus interaction in transmitting hosts, affecting the receiving hosts' virus load.

## Introduction

Human immunodeficiency virus type 1 (HIV-1) evolves under two levels of selection. On the one hand, there is within-host selection for immune escape. On the other hand, selection on the population-level acts on infectiousness and virulence. In this paper, we explore how these two levels of selection are intertwined, keeping in mind the massive heterogeneity of the hosts with respect to their cellular immune responses.

A HIV-1 infection can be separated into three phases: the acute phase, the asymptomatic phase and the symptomatic (or AIDS) phase. During the acute phase, the virus establishes high virus loads (the number of HIV-1 RNA copies per ml blood plasma) [1], until the CD4^+^ target cells are depleted [2], and adaptive immune responses start limiting viral reproduction. The virus load then drops to a semi-stable level called the set-point. This marks the beginning of the asymptomatic or chronic phase, during which the partially restored CD4^+^ T-cell count gradually drops, and at some point patients develop AIDS.

The set-point virus load (spVL) differs markedly between individuals. In untreated patients, spVL ranges from 10^2^ to 10^6^ copies/ml. The origin of this variation is an extensively researched topic, and explanations include host and viral factors. For instance, host factors incorporate the association between the set-point and the Human Leukocyte Antigen (HLA) haplotype, which is important for cellular immunity [3]–[6]. The observation that the spVL is to some extent heritable [7]–[14], suggests that viral genetic factors sway the set-point too. The exact extent of this heritability is unknown, as estimates range from 6% to 59%.

spVL is related to infectiousness and virulence. Patients with a higher spVL tend to be more infectious [15], but also develop AIDS more rapidly [16], resulting in a trade-off between infectiousness and the length of the asymptomatic phase. This life history trade-off was identified by Fraser et al. [17], and opens the door for HIV-1 adaptation with respect to transmission by means of spVL evolution. Certain spVLs (around 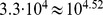 copies/ml) allow a HIV-1 strain to cause more secondary infections than strains with lower or higher set-points. A strain that establishes on average this optimal set-point should therefore become more abundant in the population. The striking observation is that, although large variation in set-points exists, most HIV-1 infected patients show a set-point close to the transmission-optimal value [17]. Moreover, mathematical models show that this adaptation can take place within realistic time scales [18], given the heritability estimates of spVL [7], and HIV-1's likely dates of origin [19], [20].

In such mathematical models, HIV-1's population-level fitness (measured in terms of the basic reproduction number 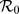) is only constrained by the life history trade-off, and environment- and mutation-induced spVL-variation. It is therefore quite intuitive that in such a model evolution leads to intermediate levels of spVL [17], [18]. The inclusion of directed within-host evolution in such models introduces an extra constraint on the population-level fitness; one which dominates the evolutionary outcome, unless within-host selection is exceedingly weak. For a homogeneous host population, this has been shown recently by Lythgoe et al. [21], and they suggest that within-host evolution of traits affecting virus load must be slow. Below we argue that ‘short-sightedness’ [*sensu* 21, 22], i.e., the life history trade-off has no apparent effect on the evolutionary outcome, can easily be understood when the host population is homogeneous. However, in a much more realistic situation where HIV-1 needs to escape from immune responses that vary markedly between individuals, the same intuition for the effect of directed within-host evolution can no longer be applied, and needs to be revised.

In this study, we explicitly incorporate such immune selection and massive host-heterogeneity with respect to immune responses in a nested epidemiological model. We investigate whether spVL evolution of HIV-1 is influenced by the virus' life history trade-off. Our model predicts that within-host immune selection has a major influence on population-wide spVL evolution. Thus, both Lythgoe's and our model predict short-sighted spVL evolution. However, we do not agree that within-host evolution must therefore be slow. Throughout the paper, we use the term ‘between-host adaptation’ for evolutionary dynamics where HIV-1's life history trade-off notably affects the evolution of spVL. The term ‘within-host selection’ refers to selection for immune escape and reversion of deleterious mutations.

At the same time, we use our model to investigate spVL heritability. We argue that high heritability can be a result of HIV-1 rapidly escaping immune responses, and the between-individual variation of these responses. We emphasize that spVL heritability caused by such a mechanism does not provide support for between-host adaptation.

## Results

### An immuno-epidemiological model

Our approach combines a caricature model for immune escape with a susceptible-infectious (SI) model for HIV-1 transmission. Both the within-host and the between-host simulations are discrete-event and individual based. The technical details are given in Methods. Here we give an intuitive exposition.

Cytotoxic T-Lymphocyte (CTL) responses are arguably important for controlling HIV-1 virus load [23], [24]. Human cells notify the cellular immune system about their proteome by presenting peptides on HLA molecules. On infected cells, a subset of these peptides originate from viral proteins. If a CTL clone detects such a foreign peptide, it can kill the infected cell, and the peptide (in its proper HLA context) is called an epitope. Not all peptides can be presented by the HLA molecules of a host, and HIV-1 can escape from CTL recognition by mutating amino acids in its peptides to prevent presentation by the host's HLA molecules [25]–[27].

Due to HLA-polymorphism, the particular subset of all peptides that can be presented by a host's HLA molecules (the binding repertoire) differs strongly between individuals [28]. In our model we incorporate this by assuming that a wild-type virus has *n* peptides that can be presented in the population. A particular host can present a subset of size *k* of these *n* peptides. During infection, we assume that mutations in the *n* potentially recognized peptides occur according to a Markov process. Some of these mutations will result in CTL escape (escape mutations). In this case, the mutant takes over the viral population in that host. Naturally, if two hosts have have a common peptide in their binding repertoires, the mutated peptide is a CTL escape for both hosts.

In line with evidence, we assume that escape mutations in HIV-1 come with a fitness cost [29], [30]. The total fitness effect of an escape mutation, resulting from immune escape and its fitness cost, must be positive before the escape mutant can replace the dominant HIV-1 strain in the host. In order to model this, we use the virus load in the asymptomatic phase as a measure for within-host fitness. An immune response causes a reduction 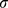 in the log_10_ virus load, and a fitness cost of any mutation reduces the log_10_ virus load by 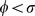. The total fitness effect of an escape mutation is then a 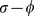 increase in the log_10_ virus load. In the simulations, we choose 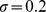 and 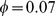 so that 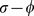 lies within estimated ranges [31], [32]. Qualitatively, our results do not depend on these particular choices for 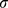 and 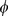, as long as 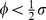 (results not shown).

Certain hosts have an efficient immune response to HIV-1. This can partially be explained by HLA-type. For instance, HLA-B*57, B*27, B*58 and B*18 are associated with a low spVL. HIV-1 is able to escape immune responses in hosts with these HLA-types, but the associated fitness costs tend to cripple the virus [25]. When such a crippled virus is transmitted to the next host, lacking the protective HLA-type, the virus load in this secondary host can remain low for a long time [29]. After a while, the crippled virus reverts the deleterious mutations, since the immune pressure causing these crippling mutations is not present in the secondary host [33]. We propose that this effect is not only restricted to known protective HLA-types, but holds more generally [e.g., see 34]. We model this similar to immune escape. As a result of immune escape in previous hosts, a viral strain may carry a number of deleterious mutations. These mutations can revert to the wild-type, again according to a Markov process.

In summary, our model for the log_10_ virus load *V* is [cf. 35]
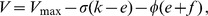
where *V*_max_ is the log_10_ virus load of a HIV-1 strain without deleterious mutations in the absence of CTL-responses (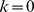), e.g., the high virus load observed in a CD8^+^ T cell depleted individual [23], [36], [37]. The integer *e* represents the number of escape mutations in a host (and hence, 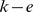 equals the number of immune responses), and *f* denotes the number of deleterious mutations. In other words, *f* equals the number of mutated peptides outside the current host's binding repertoire.

We assume that escapes and reversions appear at a rate proportional to the number of immune responses and deleterious mutations, respectively. Hence 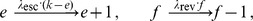
where 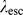 and 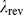 are the ‘per-peptide’ rate of escape and reversion, respectively. We will refer to 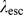 and 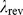 as ‘mutation rates’. Keep in mind, however, that our model of escape and reversion is quite phenomenological. The rates 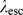 and 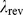 are a combination of many factors, such as the error rate during reverse transcriptase and the fixation rate. Moreover, the rates 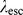 and 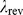 should in reality depend on the virus load. We simplify this dependence by assuming that the rates differ only between disease phases. In the acute and AIDS phase the per-peptide rates are high and in the asymptomatic phase, these rates are lower. Instead of 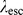 and 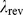, we therefore take distinct parameters 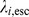 and 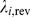 for the per-peptide mutation rate in the acute (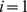), asymptomatic (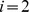) and AIDS (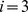) phase. We choose 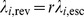 with 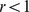, meaning that reversion is slower than escape (see Table 1 for the exact parameterization). This is in line with the assumption that the total fitness benefit of an escape mutation is greater than the benefit of a reversion (i.e. 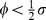).

**Table 1:** Parameters and variables of the (standard) model.

symbol	description	value	note
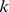	size of a hosts' binding repertoire	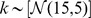 ; 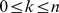	(1)
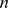	size of the union of all binding repertoires	300	(2)
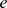	number of escape mutations	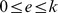	
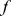	number of deleterious mutations	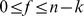	
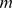	the total number of mutations	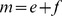	
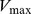	maximal 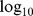 virus load	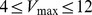	
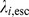	escape rate in acute ( 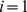 ), asymptomatic ( 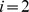 ) and AIDS ( 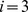 ) phase	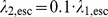 ; 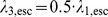 ; 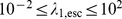	(3)
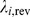	reversion rate during disease phase 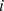	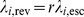 , where 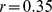	(4)
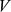	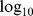 virus load	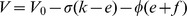	(5)
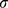	decrease in 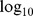 virus load due to one immune response (without the fitness cost)	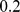	(6)
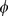	fitness cost of a mutation	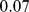	(6)
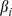	infection rate during disease phase 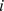	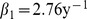 ; 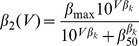 ; 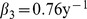	(7)
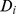	(mean) duration of disease phase 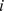	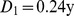 ; 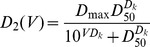 ; 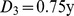	(7)

Notes: (1) 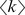 is chosen larger than observed numbers of immune responses [25], [27], [43], since we predict that viruses have escape mutations at infection, and do not escape all CTL responses. 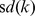 is chosen to get reasonable variance in spVL, while limiting individuals with a very small binding repertoire. (2) About 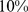 of all possible peptides from HIV-1's proteome of 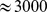 a.a. (3) During the chronic phase, the escape rate slows down markedly [27], [60], hence we take 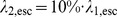. The AIDS phase is sometimes preceded by escape from critical immune responses [75], and modeling suggests that escape rate speeds up towards the late disease phase [46]. Therefore we set 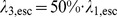. (4) Both reports on fast [76] and very slow [77] reversion exist. We choose 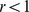 in the order of magnitude of the ratio fitness cost and escape benefit. (5) The model for virus load was taken from [35]. During the acute phase, 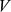 merely represents the virus fitness. (6) The magnitude 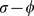 is chosen to be in estimated ranges [31], [32]. Since escape appears to be faster than reversion, we choose 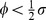. Although several studies find that a CTL response to Gag gives a 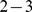 fold higher fitness cost than 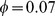 [29], [32], we take 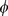 as an average fitness cost. (7) The parameters 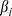 and 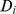 were taken from [17]. The parameters for the Hill functions 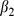 and 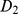 are: 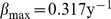, 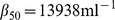, 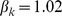, 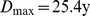, 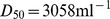, 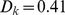.

**Figure 1:**
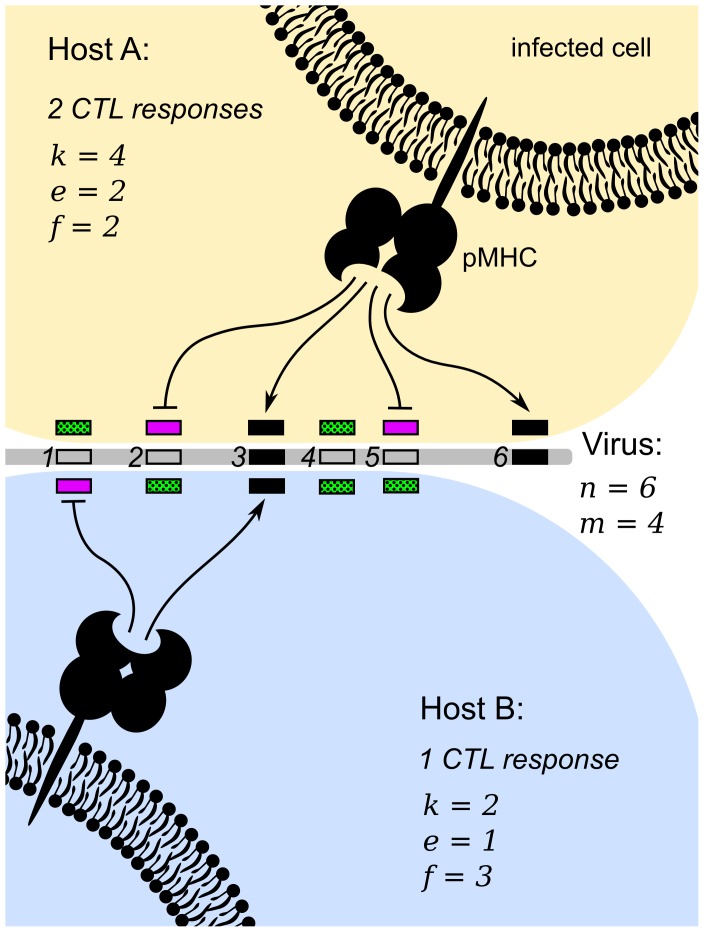
The phenotype of a virus differs between hosts, depending on the hosts' HLA haplotype. The virus in the figure has 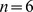 potential epitopes (the rectangles), of which 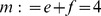 have a mutation (the open rectangles). ‘pMHC’ denotes the peptide-HLA complex. (Host A) Host A's HLA molecules can not bind peptides 1 and 4 (neither the wild-type, nor the mutant), but they can bind the wild-type of peptides 2 and 5. Thus, the purple rectangles denote immune escape mutations and the green (dotted) rectangles represent deleterious mutations. Since peptides 2 and 5 are mutated, they are escape epitopes in host A. The HLA molecules of host A can bind peptides 3 and 6, and hence peptides 3 and 6 are the epitopes for host A. During the infectious lifetime of host A, epitopes 3 and 6 may escape, and the mutated peptides 1 and 4 may revert to the wild-type. (Host B) The HLA molecules of host B bind less peptides of the wild-type virus (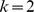) than host A (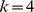); host B mounts a single CTL response against peptide 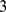. The HLA molecules of host B can also bind the wild-type of peptide 1, but this peptide is mutated, and hence peptide 1 is an escape epitope in host B. During host B's infection, epitope 3 may escape, and peptides 2, 4 and 5 may revert to the wild-type.

As mentioned earlier, a HIV-1 strain infecting a new host carries a history of mutations acquired in previous hosts [29], [38]. In the context of the new host, many of these mutations will not be beneficial. Some of them may be advantageous, because HLA molecules can share epitopes [39], [40], and individuals share HLA molecules. To keep our model simple, we assume that a random host's binding repertoire is a random subset of size *k* of the set of all *n* possible HIV-1 epitopes. In reality, HLA haplotypes, and hence binding repertoires, are less regularly distributed. However, our simpler distribution provides us with the advantage that we only have to keep track of the *number* of mutated peptides. Namely, when a host transmits a virus with *e* escape mutations and *f* deleterious mutations (denoted as an 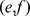-virus), then in the secondary host the virus will have phenotype 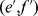 with 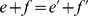. We find the number of escape mutations *e*′ by choosing a new random binding repertoire of size *k*′. Since every peptide is part of the new binding repertoire with equal probability, the number of *a priori* escape mutations is drawn from the hypergeometric distribution (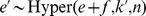). An example of how a virus' phenotype can differ between hosts is given in Figure 1. By default, we choose 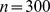 and 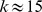 (see Table 1), such that about 10% of HIV-1's peptides can serve as an epitope. The number *k* is chosen such that hosts have a realistic number of responses, also when many of the *n* peptides are mutated.

We model the three phases of a HIV-1 infection based on Fraser et al. [17] and Hollingsworth et al. [41]. The acute phase has a fixed length *D*_1_, and in this phase individuals have a fixed infectiousness *β*_1_. After *D*_1_ years, the asymptomatic phase starts and infectiousness *β*_2_(*V*) and the average length of the asymptomatic phase *D*_2_(*V*) depend on the virus load *V*. The functions *β*_2_ and *D*_2_ are Hill functions with coefficients as estimated by Fraser et al. [17]. When the asymptomatic phase ends, the AIDS phase starts. This AIDS phase has, similar to the acute phase, a fixed length *D*_3_ and fixed infectiousness *β*_3_. We do not incorporate any correction for serial monogamy on infectiousness.

**Figure 2:**
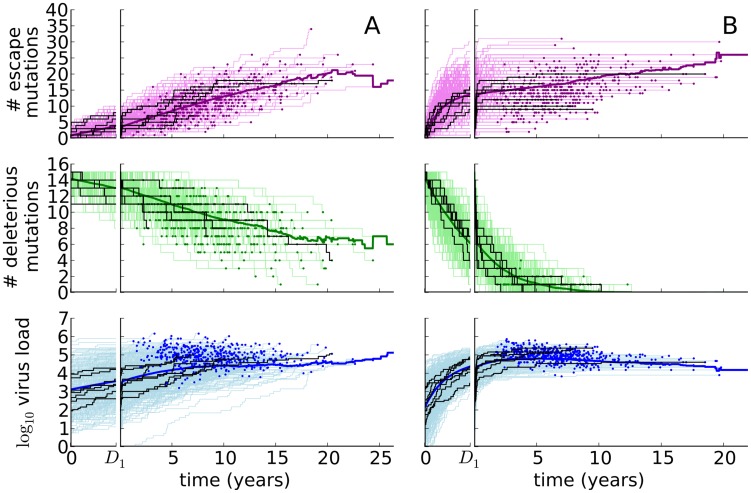
The within-host model for immune escape for different mutation rates. The graphs show the number of escape mutations (purple, top), the number of deleterious mutations (green, middle) and the virus load (blue, bottom). The mean number of mutations or virus load (the heavy lines) is based on 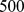 simulations (the thin step-wise lines). The dots indicate that a host died. All infections start with a virus with 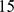 mutations. The acute phase of the infection (the first 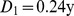) is displayed magnified on the left of each plot, and a couple of simulations are highlighted in black. (A) The escape rate equals 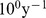, and 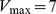. (B) The escape rate equals 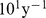, and 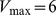. Other parameters are listed in Table 1.

As an illustration of the within-host model, we have simulated a large number of within-host processes for two different parameter settings (Figure 2). Stochasticity and host-heterogeneity cause large variation in the within-host evolution of the virus (the thin step-wise lines in Figure 2). As deleterious mutations are reverted and CTL responses are escaped, the virus load increases during the infection. If the mutation rate is high, almost all escapes happen during the acute phase of the infection. The cohort-average virus load (the heavy blue line in Figure 2, bottom panels) can then even decrease, since individuals with a high set-point develop AIDS more rapidly. When these fast progressors die, we exclude them from the calculation of the cohort's mean virus load. Notice that during the acute phase, the variable *V* does not reflect the peak virus loads observed in patients, but is merely a measure of the virus' fitness.

For the between-host model, we explicitly model a population of infected individuals (of size *I*), and assume a frequency-dependent contact process with susceptibles [42]. Super-infection and co-infection are ignored. We keep the total population size (*N*) constant, and only keep track of the susceptibles' number (*S*). Because of within-host evolution, an individual may transmit different viral strains during the course of an infection. When the virus load increases due to within-host adaptation, the infection rate also increases. We verified that a model with a non-constant population size does not give different results (not shown).

Since in our model the virus load can increase during the asymptomatic phase, we need to specify what we mean with set-point virus load. We define the spVL (in log_10_ scale) as the geometric average of the log_10_ virus loads in the asymptomatic phase, i.e., 
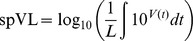
, where the integral is taken over the chronic phase, which lasts *L* years, and *V*(*t*) denotes the virus load at time *t*. We often write 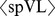 to indicate the population-wide arithmetic average spVL. Bracket notation is also used for other population-wide averages.

### For realistic mutation rates, selection for immune escape dominates HIV-1 virulence evolution

**Figure 3:**
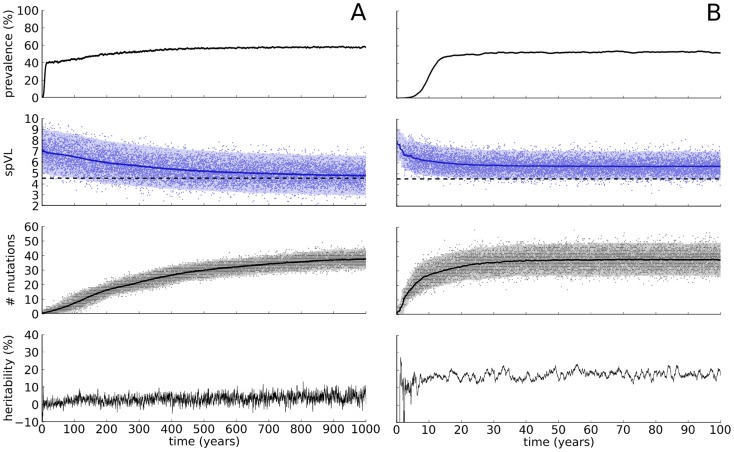
Two simulations of HIV-1 epidemics with two different mutation rates. The parameters are as follows: The maximal virus load equals 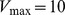, and the population size equals 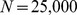. (A) The escape mutation rate in the acute phase equals 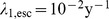. (B) The escape mutation rate in the acute phase equals 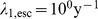. The other parameters are listed in Table 1. The simulations were started with 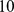 infected individuals that were infected with a virus with 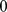 mutations. The heavy lines in the graph of the set-point (spVL) and the number of mutations (# mutations) denote the population-wide average, i.e., 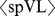 and 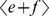, respectively. The light bands denote the 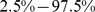 percentiles, and the dots indicate the spVL of the receiver of a transmission couple (spVL) and the number of mutations of the transmitted strain (# mutations). In the graphs of the spVL, the dashed black line indicates the mean set-point that maximizes the transmission potential of HIV-1.

When we choose the mutation rate low and run the agent-based model, the mean spVL converges to 4.52log_10_ copies/ml; the value optimal for transmission (Figure 3A). However, this takes many centuries, depending on the maximal virus load *V*_max_ and the initial number of mutations. By increasing the mutation rate, we make the evolutionary dynamics faster, but lose between-host adaptation (Figure 3B). In fact, the mean spVL is approximately 1.3 log_10_ copies/ml higher than 4.52. By keeping the mutation rate equally high, but lowering *V*_max_, the HIV-1 quasi-species can be given a population-level fitness (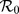) that is about 17% higher than what is reached in Figure 3B. Apparently, selection for spVL values that are optimal for transmission is overruled by within-host selection at high mutation rates.

**Figure 4:**
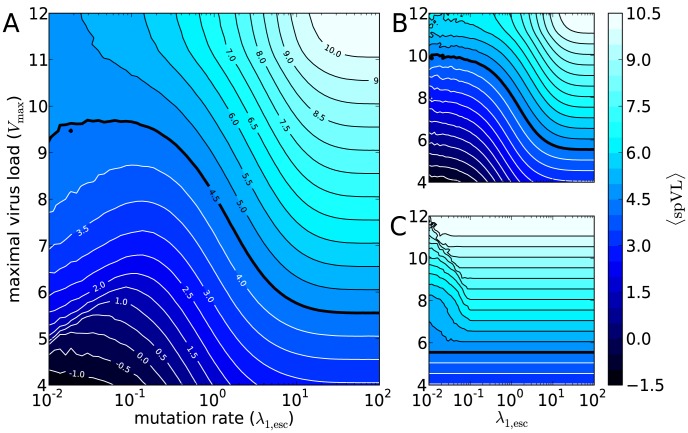
Exploration of the parameter space in three scenarios. The contours show the mean set-point virus load in the population-level equilibrium. The heavy black line indicates the graph of 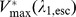, i.e., the value 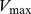 for which 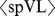 is optimal for transmission, given the mutation rate 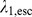. (A) The standard model: A peaked TP and a heterogeneous host population. (B) Control 1: A flat TP and a heterogeneous population. (C) Control 2: A peaked TP and a homogeneous population.

Both simulations in Figure 3 are approaching different steady states. Thus, to investigate between-host adaptation further, we now look at the properties of the model in population-level steady state for many different parameter combinations (Figure 4). To make the analysis computationally feasible, we stochastically approximate the next-generation matrix (NGM, see Methods). We fix all parameters except for the mutation rates (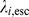 and 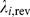), and the maximum virus load (*V*_max_). We keep the ratios 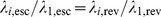 and 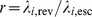 between the mutation rates constant (see Table 1 for the parameters chosen). Apart from the standard model described above, we also consider two modifications that serve as controls. In the first control, we take out the effect of population-level selection for transmission. In the second control, we make the population homogeneous.

#### The standard model

**Figure 5:**
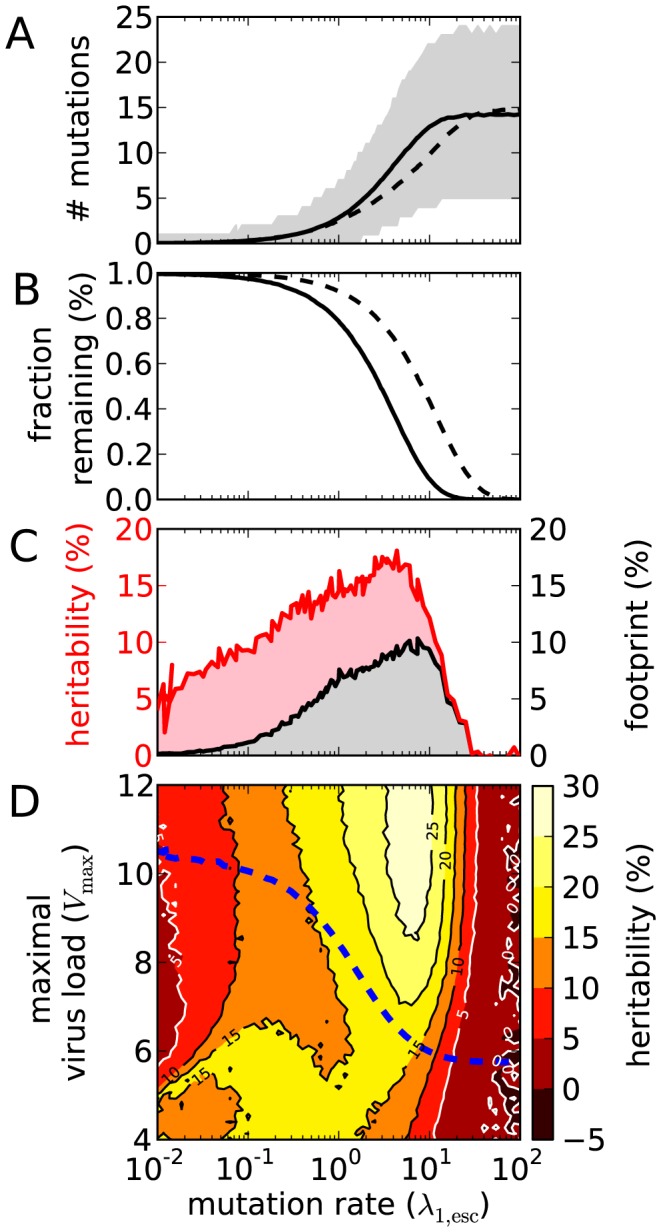
Heritability of set-point and the number of mutations during the acute phase. (A) The average number of escape (solid) and deleterious (dashed) mutations at the end of the acute phase for different 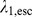. The resulting graph is barely dependent on 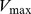 within the range 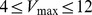 (not shown). However, we choose 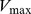 such that the mean set-point virus load (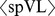) equals 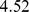, cf. the blue, dashed line in panel D (this also holds for panels B and C). The gray band indicates the 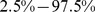 percentiles for the number of escape mutations in the acute phase. (B) The mean fraction of immune responses (solid) and deleterious mutations (dashed) that remain after the acute phase. The resulting graph is barely dependent on 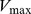 within the range 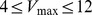 (not shown). (C) Heritability as a function of the mutation rate (upper red line). The black line below corresponds to the contribution of the immunological footprint to heritability, as estimated with the SEM. (D) Heritability of set-point virus load for different combinations of 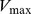 and 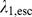 for the standard model in steady state. The blue, dashed line indicates the contour where the 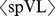 equals 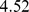.

In this model, the population is heterogeneous (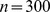 and 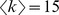), and virulence and infectiousness are taken from Fraser et al. [17], as described above. We refer to the resulting transmission potential (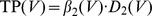) as ‘peaked’, because a single spVL value exists at which the number of secondary infections caused by one infected individual is maximal. Considering the population-level steady state for very high mutation rates can give us a measure of between-host adaptation. When we choose the escape rate in the acute phase (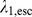) close to 10^2^y^−1^, the virus will escape all immune responses, and revert all deleterious mutations acquired in previous hosts, during the first few weeks of the infection (Figure 5B, black graphs). The population-average spVL will therefore tend to 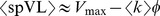. In Figure 4A this relation is visible when 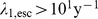 from the equidistant contours of the set-point in equilibrium. When we replace *V*_max_ by, say, *V*_max_+0.5 and we observe that 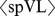 changes into 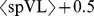, then the virus is not capable of between-host adaptation. In our parameter space exploration, this isometric dependence of 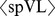 on *V*_max_ can not only be observed for unrealistically high, but also for intermediate mutation rates.

Notice that our parameter space exploration can be regarded as a sensitivity analysis. For a fixed escape rate 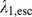, a value 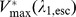 exists such that 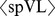 is optimal for transmission. By varying *V*_max_ around the value 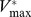, we can study the sensitivity of 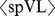 with respect to *V*_max_. In Figure 4 the graphs of 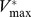 are given by heavy black lines.

A different picture emerges for low mutation rates (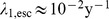). The contours are no longer equidistant, indicating that the steady state, the result of mutation and selection, is less sensitive to changes in *V*_max_. The absence of *V*_max_-sensitivity is most noticeable when 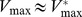. This suggests that the virus is able to adapt on the level of the population (between-host adaptation). We will confirm this using Control 1 below. As mentioned before (Figure 3), between-host adaptation takes many centuries for small mutation rates. The within-host process for this parameter regime is extremely slow.

By considering the number of escape mutations in the acute phase, we can get insight into what parameter regime is realistic for HIV-1. Several studies show that the number of escape mutations in the first months after infection varies among patients, and lies between 1 and 10 [25], [27], [43]. This suggests that for escape rates 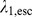 to be considered realistic, they must be in the range 10^−1^ to 10^1^y^−1^ (see Figure 5A). For these intermediate mutation rates, we see a strong effect of host-heterogeneity. Host-heterogeneity and subsequent infections that require new escape mutations, account for the accumulation of deleterious mutations, since deleterious mutations are not lost at a fast enough rate. The virus' inadequacy to fully adapt to individuals during infection decreases the within-host virus load. We will further justify this with Control 2 below. However, the lack of perfect adaptation to individuals' immune systems does not noticeably facilitate between-host adaptation. In the regime of realistic CTL escape rates (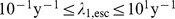), spVL evolution is not driven by the life history trade-off, as can be seen from the isometric relation between *V*_max_ and 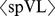 (Figure 4A).

#### Control 1, eliminating population-level selection

By comparing the standard model with a model where no between-host adaptation is possible, we can study the impact of the peaked transmission potential. We eliminate selection for transmission by scaling each host's infectiousness such that the expected number of secondary infections during an entire infectious lifetime equals the same constant for all individuals. The transmission potential will therefore be ‘flat’. To make this precise, let *β*(*t*) denote an individuals infection rate at time *t* (depending on disease phase or virus load). We now want to make sure that each individual is expected to infect 2 new individuals (in a fully susceptible population). To achieve this, we replace *β*(*t*) with 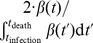. Here *t*_infection_ and *t*_death_ denote the time of infection and death, respectively. Notice that we do allow for variation in virulence; the length of the infectious period (*t*_death_−*t*_infection_) is equally dependent on virus load as in the standard model. Hence, we eliminate selection for transmission without altering the within-host process.

Figure 4B shows the mean spVL in steady state for the flat TP and a heterogeneous population (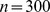, 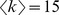). The evolutionary outcomes for control 1 and the standard model are nearly identical when the escape rate 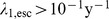. This confirms that the virus is not capable of between-host adaptation when mutation rates allow for a realistic number of escape mutations during an infection. For extremely low mutation rates (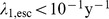), we see a difference between the model with a flat and a peaked TP, confirming that between-host adaptation relies on low mutation rates.

In the slow mutation regime, optimizing the life-history trade-off can be accomplished in two ways. If the virus were to experience a flat transmission potential, then a quasi-species' spVL distribution and the number of accumulated mutations would only be determined by the rate of escape and reversion, and the heterogeneity of the host population. If the same species starts evolving under the influence of a peaked transmission potential, then the number of mutations might either decrease, resulting in a lower fitness cost, and a higher 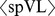 (e.g., when 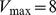 and 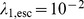), or the number of mutations might increase, resulting in a lower 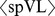 (e.g., when 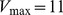 and 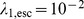). Notice that mutations always arise as CTL escapes in an individual, but that such a mutation is most often deleterious in the other hosts.

#### Control 2, the homogeneous case

The effect of HLA-polymorphism can be studied by considering a model without host-heterogeneity. In the models with heterogeneous host populations, we assigned upon infection of a new individual a random binding repertoire, i.e., a random subset of size *k* of the virus' *n* potential epitopes. For this control, we assign to every host exactly the same binding repertoire. As a consequence, escape mutations remain beneficial after infection of a new host. Notice that in our model deleterious mutations always originate from escape mutations in earlier hosts with a non-identical binding repertoire. Therefore, deleterious mutations are purged from the population.

Figure 4C shows the mean spVL in steady state in case of a homogeneous host population (with a peaked TP). For a wide range of mutation rates, the virus manages to escape all immune responses. As a consequence, there is no room for population-level adaptation with respect to transmission, which is visible from the equidistant 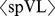-contours, and the isometric relation between *V*_max_ and 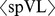. If we compare Figure 4A and C at intermediate mutation rates (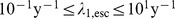), we indeed see that host-heterogeneity lowers the set-point drastically.

For small mutation rates (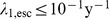) we observe a threshold, which depends on *V*_max_ and 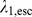. For mutation rates below this threshold, viruses evolve that do not escape all immune responses. When the mutation rate is small enough, escape mutations are rare. Viral strains that do escape yet another immune response will establish a higher set-point virus load. These escape mutants are then out-competed on the population level by strains that are better recognized by CTLs, because the life-history trade-off favors a lower set-point.

**Figure 6:**
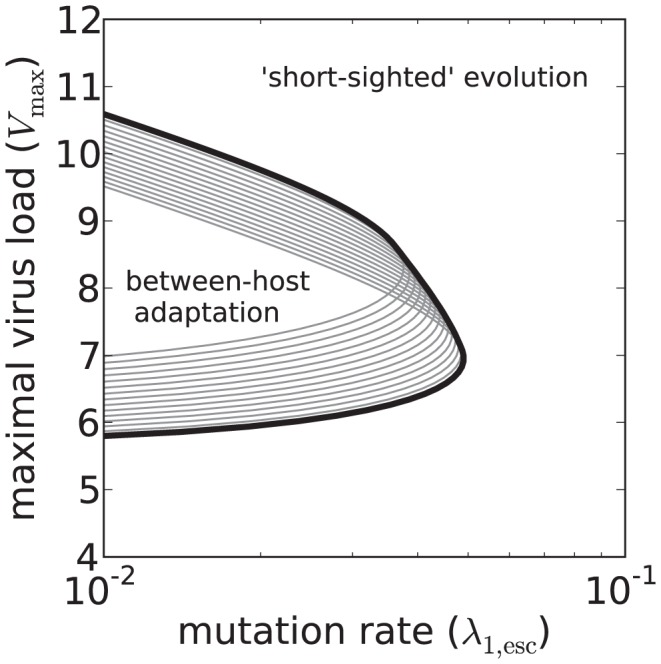
The bifurcation in the homogeneous model. For each 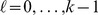 a thin gray line indicates the curve 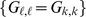. The heavy black line separates the region of the parameter space (between-host adaptation) where 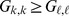 for all 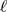.

The above mentioned threshold can be better understood by studying the NGM. The stochastic model can be simplified so that a mathematical analysis is possible. The threshold for the homogeneous model can be described in terms of the eigenvalues of the NGM and is caused by a transcritical bifurcation (see Methods). The heavy black line in Figure 6, which we find with the mathematical analysis, gives the location of the bifurcation. This line separates the parameter space into a region where between-host adaptation is possible, and where it is not. The line coincides with the apparent threshold that can be observed in Figure 4B.

### Immune escape causes heritability of set-point virus load

Between-host adaptation is only possible if spVL is inherited from one person to the next. If the speed of within-host adaptation is intermediate or fast, our model does not predict population-level adaptation for transmission. Here we show that the absence of between-host adaptation is not due to lack of spVL heritability (*h* ^2^, see Methods). To this end, we compute heritability during an epidemic (see Figure 3, bottom panels), and in the steady state of the (standard) model for many different parameter combinations (see Figure 5C).

During a simulated epidemic, we use all transmissions that take place within a time span of a year to compute heritability. This means that the sample size for computing heritability equals the (yearly) incidence. The median incidence in the simulation with a low mutation rate (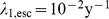, Figure 3B) equals 2335 yearly infections (2.5th, 97.5th percentiles: [1839,4085]). For the simulation with a faster mutation rate (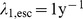, Figure 3A), more virulent viruses evolve, and the median incidence equals 3209 infections per year (2.5th, 97.5th percentiles: [241,3586]). Even with these large sample sizes, heritability fluctuates substantially. In Figure 3B the median of *h* ^2^ is 3.12% (2.5th, 97.5th percentiles: [−1.31,8.29]), and in Figure 3A the median of *h* ^2^ is 17.1% (2.5th, 97.5th percentiles: [10.8,21.8]). The rapid fluctuation in *h* ^2^ might explain why different experimental studies to HIV-1 spVL heritability that use transmission couples [7], [11]–[14] often give quite varying results [cf. 9]. The NGM approach allowed us to produce an even larger number of transmission couples, and hence, to estimate heritability more accurately. Overall, heritability lies between 0% and 30%, and is 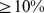 for realistic parameter combinations.

In our model, one can think of two mechanisms that cause heritability. The first mechanism applies when mutation rates are not too high. If variation in the number of mutations exists and the mutation rate is low, the spVL of transmitting and recipient hosts are correlated, although this correlation will not be perfect due to the variation in the breadth of the immune response (*k*). If the mutation rate increases, viruses adapt to their host more rapidly and, according to this first mechanism, the correlation vanishes.

The second mechanism is related to transmission of crippled viruses. If a host controls the infection well because of a broad immune response, the virus will escape more CTL responses and, when transmitted, becomes crippled in the average new host. In the primary, controlling host, the set-point virus load is low due to good initial immune responses and the virus' fitness cost of escape, and in the average secondary host the virus load will again be low due to the high number of deleterious mutations. *Vice versa*, in hosts with a narrow immune response, transmitted strains will have few new escape mutations and this will lead to few deleterious mutations in the recipient.

We can most clearly see the effect of the second mechanism when both mutation rate and *V*_max_ are high (the contour 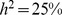 in Figure 5C). In this part of the parameter space, most immune responses are escaped in the acute phase (cf. the solid graph in Figure 5B). Rapid escape causes variation in the number of deleterious mutations in the transmitted virus, because the size of the binding repertoire (*k*) varies among individuals. However, when 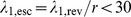, not all deleterious mutations can be reverted in the acute phase (cf. the dashed graph in Figure 5B). For high *V*_max_, the asymptomatic phase is short, resulting in few reversions during this phase and a ‘footprint’ of the transmitting host's immune responses on the receiving host's spVL [11]. Notice that the second mechanism does depend on the reasonable assumption that reversion is a slower process than escape (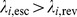 and 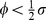, not shown), and that the size of the binding repertoire (*k*) differs between individuals.

**Figure 7:**
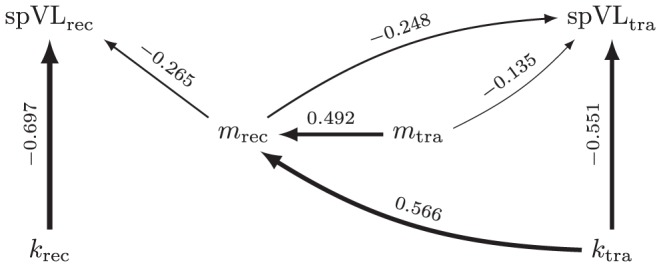
The structural equation model (SEM) used for quantifying the immunological footprint. Shown is a directed, acyclic graph (DAG) representing the SEM. The arrows indicate dependencies between the variables. The numbers above the arrows are the fitted weights (all highly significant: 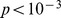), and the size of these weights is also represented by the thickness of the arrows. The data for this example comes from a simulation of the standard model, with 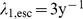 and 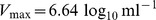.

As the above evidence for the second mechanism—or ‘footprint effect’ as we like to call it—is only circumstantial, a quantification of this mechanism is needed. To quantify the footprint effect we analyze the simulations using a structural equation model (SEM). The model estimates heritability, and takes the fitness costs (*m* = *e*+*f*) and breadth of the immune response (*k*) into account. Heritability of spVL is the sum of two effects; one mediated by viral fitness, and the other by the breadth of the immune response of the transmitting host. Figure 7 shows a graphical representation of the model, and details of the analyses are given in the Methods section.

For realistic parameter values, approximately half of the observed heritability is due to the footprint effect (Figure 5C). When we lower the rate of escape, the footprint effect, and therefore also the heritability, decreases. On the contrary, when within-host evolution is extremely fast, almost all of the heritability is due to the footprint effect, although the total heritability decreases.

### Host heterogeneity and spVL heritability: A model-based prediction

Our model predicts that heritability of the set-point virus load and host-heterogeneity are related. When within-host evolution is fast enough, approximately half of the observed heritability may be explained by the immunological footprint. Also, when we lower heterogeneity in our model, heritability decreases.

An intuitive measure for heterogeneity in the host population is the expected similarity of hosts’ binding repertoires. This tells us how much adaptation to one host remains beneficial in the next. As a measure of the similarity of two binding repertoires *K*_1_ and *K*_2_ (of size *k*_1_ and *k*_2_, respectively) we use the Jaccard index 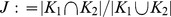, the overlap between binding repertoires, divided by the the number of (wild-type) epitopes that at least one of the hosts can recognize.

**Figure 8:**
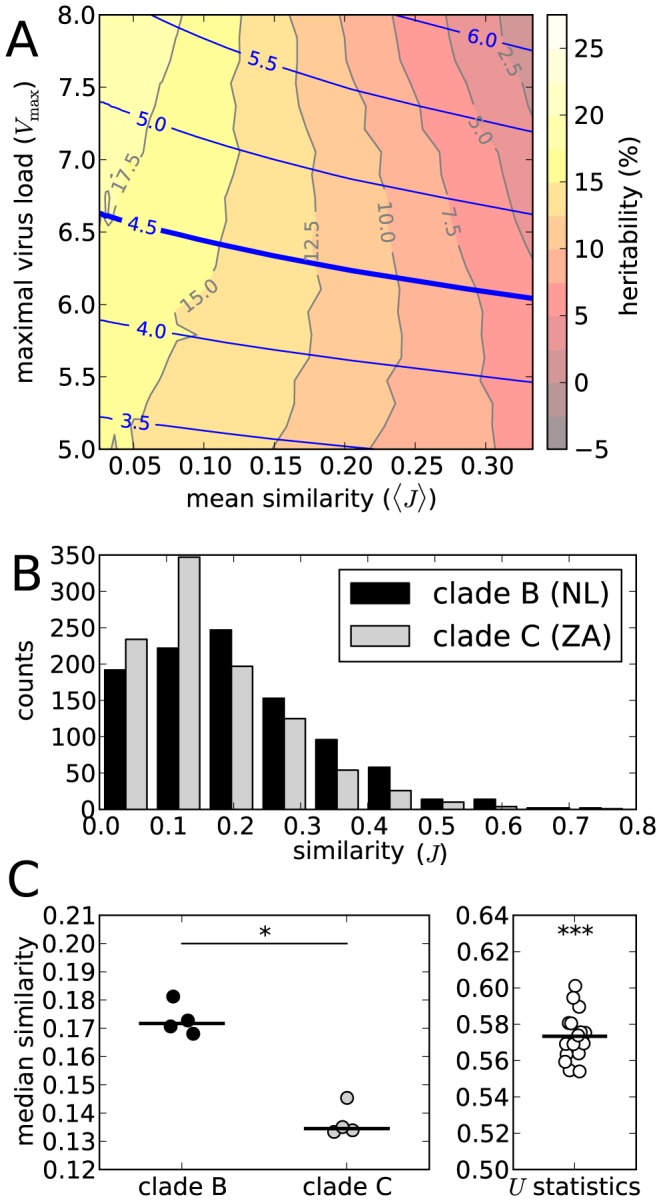
Host-heterogeneity and heritability. (A) The panel shows a contour plot of heritability (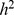, gray lines, red/yellow faces) as a function of the maximal virus load (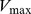) and the expected similarity between binding repertoires (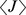). On top of the heritability contour plot, the blue lines indicate the contours of 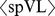. The heavy blue contour corresponds to the transmission-optimal 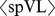. (B) Distributions of the overlap between pairs of binding repertoires. The black bars correspond to European HLA-haplotypes and a clade B virus (sampled in the Netherlands). The gray bars correspond to Sub-Saharan HLA-haplotypes and a clade C virus (sampled in South Africa). The distributions were simulated by sampling a 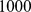 HLA-haplotype pairs. (C) Statistics on the sampled distributions as in panel B. The left panel shows the medians of the similarity distributions for 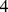 strains representative of clade B (black dots) and clade C (gray dots). The difference is significant (Mann-Withney 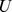-test, 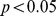, *). The right panel depicts the 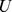-statistic for all clade B and clade C pairs. The mean of the 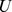-statistics is significantly larger than 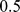 (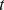-test 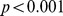, ***).

Figure 8A shows the relation between the expected similarity between hosts (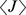) and the heritability of the set-point (*h* ^2^). We modulated heterogeneity by varying *n*, the total number of potential epitopes, between 30 and 300, corresponding to low and high host-heterogeneity, respectively. The mutation rate 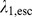 equals 3y^−1^, such that the number of escape mutations during the acute phase lies within a realistic range. The figure shows that heritability indeed decreases when the population becomes more homogeneous, which indicates that high heritability relies on host-heterogeneity.

HLA-heterogeneity differs between human populations. If our model prediction holds, then this variation could affect the heritability of the set-point measured in these populations. An unpublished study by Hodcroft et al. [44], shows that heritability in measured for HIV-1 clade C in a Sub-Saharan African population is higher than heritability for HIV-1 clade B in a European cohort [10] (30% vs. 5.7%). Keeping our model in mind, we are able to understand this, if the European population with respect to clade B, is less heterogeneous than the Sub-Saharan population with respect to clade C.

Using the peptide-MHC binding predictor NetMHCpan [45], we compared the two populations and circulating viruses (see Methods). Again, we measured heterogeneity in terms of similarity between binding repertoires. We sampled from the HLA-haplotype distributions of the European and Sub-Saharan populations, and calculated how similarity (again measured in terms of the Jaccard index) within these populations is distributed. Figure 8B shows two of these distributions. The black bars correspond to the European population, and the gray bars to the Sub-Saharan population. Although small, these populations do show a difference in heterogeneity: The Sub-Saharan population is more heterogeneous than the European population, as European binding repertoires tend to be more similar. The difference in heterogeneity is statistically significant (see Methods and Figure 8C).

## Discussion

In this paper, we model HIV-1 transmission and within-host adaptation by means of immune escape in a HLA-heterogeneous host population. In comparison to what data suggests, we do not find that HIV-1's life history trade-off determines or influences spVL evolution. For realistic mutation rates, the evolutionary outcome is mostly determined by within-host selection for escape and reversion. Without HLA-heterogeneity, viruses would evolve to be within-host optimal in every host. Due to HLA-polymorphism, however, deleterious mutations accumulate, and the environment changes at each transmission. This causes virulence to evolve to intermediate levels for most hosts. Incomplete adaptation at the individual level is not exploited by the virus in order to improve it's transmission potential. Although set-point virus loads are expected to be lower in a heterogeneous population, spVL evolution remains short-sighted. As we will point out below, our model is limited in the sense that we only incorporate immune escape and reversion as a means for within-host and between-host adaptation. Nevertheless, since population-level adaptation does occur when within-host adaptation is slow, the model's limitations do not necessarily revoke our conclusions.

In our model, we do find that spVL is heritable, even when the mutation rate is high. spVL heritability is needed for between-host adaptation. However, for realistic mutation rates, high heritability, as measured using transmission couples, is over-estimated; it mostly results from a ‘footprint’ left by the transmitter's immune system on the receiver's spVL. This novel explanation calls the validity of the use of high heritability as support for between-host adaptation in question. During real HIV-1 infections, immune escape sometimes requires compensatory mutations. Such escape variants need more time to revert to the wild-type in hosts lacking the escaped CTL response [46]. Such a mechanism is not incorporated in our model, but is likely to cause even higher heritability compared to what we find. Given previous results on the effects of transmitted CTL escape mutations on a receiver's virus load [29], [33], and the sharing of HLA alleles [11], [47], we think the footprint effect provides a sound explanation for the experimentally observed high heritability of the set-point. Importantly, if this explanation were to be found true, and if spVL evolution and heritability are indeed strongly influenced by CTL escape, reversion and compensatory mutations, finding SNPs in HIV-1's genome that control spVL might be a fool's errand, unless this pursuit would be restricted to known CTL-epitope sites.

Our claims concerning the footprint effect, and the dependency of heritability on host-heterogeneity are not just speculative. We show that the model can make testable predictions, and we give an example of how such a test can be performed, i.e., by comparing host-heterogeneity in different human populations. In our example, we compared Sub-Saharan and European populations with respect to the viruses circulating in these populations, and showed that host-heterogeneity is higher in the African population, which is consistent with our novel explanation, and estimates of the heritability in these populations. Of course, we would not suggest that this isolated finding is evidence for the footprint effect, although we do want to stress that heritability estimates are expected to be correlated with host heterogeneity. Moreover, the heritability estimates that were used in this example were obtained using a phylogenetic analysis [10], [44], while our explanation only holds for studies that use transmission couples. In future work, we plan to investigate whether an immunological footprint can also affect heritability that has been estimated using phylogenies or pedigrees.

Intuitively, the fact that within-host adaptation overrules between-host adaptation can be understood by considering that many viral generations separate the founding virus and a transmitted strain, while transmission only takes one generation. In the homogeneous model, this results in full within-host adaptation (throughout the population all epitopes are escaped), except when within-host adaptation is extremely slow. This result was also shown recently by Lythgoe et al. [21].

The intuition mentioned above works best for homogeneous populations. Adaptations to a primary host are beneficial again in a secondary host, and if within-host adaptation is fast, this leads to population-wide within-host adaptation and not between-host adaptation. This part of the intuition fails for a heterogeneous host population, where within-host adaptations in the form of immune escapes, are most likely not beneficial in the next host. Therefore, one could argue that homogeneity obstructs between-host adaptation. Here, we attempt to remove that obstruction by adding host-heterogeneity to a multi-level HIV-1 model. We find that in a heterogeneous population, HIV-1 also fails to evolve a mean spVL that maximizes the transmission potential, as shown by our sensitivity analysis and controls. Of course, when we make within-host adaptation trivial by choosing a very low mutation rate, population-level adaptation occurs.

Apparently, host-heterogeneity does not solve the within- versus between-host adaptation paradox. Our models tell us that within-host adaptation overrules between-host adaptation, and yet HIV-1 appears to have adapted with respect to the life history trade-off [17], or at least is evolving its mean spVL towards the value that maximizes the transmission potential [48]. Several mechanisms that can serve as a solution for the paradoxical observation have been proposed [49]–[51].

One of these mechanisms is referred to as ‘store and retrieve’ [49]. It is hypothesized that latently infected memory CD4^+^ cells occasionally produce virus, and that these virions are preferentially transmitted. Preferential transmission is backed up by the observation that evolutionary rates are higher at the within-host than at the between-host level [52], and recently by a very interesting study into HIV-1's transmission bottleneck [53]. However, transmission of CTL escape mutants within transmission couples [29], and even the spread of CTL escape mutants through populations has been observed [38], [54]–[57]. These observations indicates that ‘store and retrieve’ is not absolute, and in order for this mechanism to solve the paradox, we expect it to rely on getting the population-level evolutionary rate below a threshold; one which may not be reached. This premise could limit the robustness of the ‘store and retrieve’ model. Furthermore, when the population-level evolutionary rate is slowed down because of a mechanism like ‘store and retrieve’, the rate of between-host adaptation is also decreased, which could conflict with the short time scales at which adaptation must have been taking place for HIV-1 [18], [20].

Another possible mechanism is a heritable viral trait that influences spVL, but that is not under within-host selection. This was recently examined by Hool et al. [50]. An example of such a trait could be target cell activation rate [58], [59]. In short, if a viral trait influences target cell activation, and a mutant strain manages to increase the activation rate, then this additional activation is a ‘common good’ for the entire within-host viral population (activated cells produce more virions). Hence, the mutant does not have an advantage and will not be preferentially selected. Drift creates within-host variation in activation rate, and the transmission bottleneck leads to variation of the target cell activation rate at the population-level. This hypothesis could be challenged by other traits that affect spVL, since these may still be under within-host selection, and are likely to interfere with the within-host neutral one.

We finish with a novel suggestion for solving the paradox, one which is based on our modeling formalism, and was recently also put forward by Fraser et al. [51]. One point of criticism on our model could be that we limit the evolutionary capabilities of our *in-silico* viruses. Strains can only evolve their number of deleterious mutations in order to approach population-level favorable spVLs. Unfortunately, in the current framework, it is not sensible to allow for mutations in other parameters, in particular *V*_max_, since then *V*_max_ would only increase during within-host evolution, and hence, during the course of the epidemic. This is because we assume that no two strains simultaneously reside a single host, and that mutants with a higher fitness go to fixation rapidly. In reality, fixation of mutants within a host can take a considerable amount of time [60].

An obvious—but technically challenging—fix for this problem is to abandon the assumption that the within-host evolutionary dynamics is memoryless, and allow for multiple mutants to compete for fixation, i.e., allow for clonal interference [61]–[65]. These mutants can then carry negative fitness effects (e.g., *V*_max_ decreasing mutations) along with beneficial escape mutations or reversions (genetic hitchhiking). Additionally, mutants with a small *V*_max_ increasing effect, but that are otherwise equal to the wild-type, may have a long fixation time and can easily be out-competed by, e.g., escape mutants. This makes within-host *V*_max_ evolution more selectively neutral, and hence more sensible in our model. In future work, we aim to test if these speculations are valid, and whether a more detailed within-host fitness and selection model can unify within-host evolution and population-level adaptation.

## Methods

Our full model is a two-level individual and discrete-event based simulation, based on the Sellke construction [66]. The Sellke construction generalizes the Gillespie algorithm, by allowing for non-exponentially distributed waiting times. We need this generalization to allow for realistic non-exponential distributions of the length of the asymptomatic phase, as estimated earlier [17]. Events in our simulation occur at particular points in time, which determines the order of these events. If an event takes place, this may alter the state (e.g. the number of susceptible individuals, or the virulence) and this influences the moments and order at which future events take place. The model was coded in C++ and analyzed using Python and R. The code has been made available as an electronic supplement (File S1).

The agents and events that are described explicitly in our model are listed in Table 2. In order to determine what the next event will be and when it takes place, we need to know how to compute waiting times.

**Table 2:** Agents and events in the model.

	Agent	Events
*within-host level*	virus	escape mutation, reversion
	disease phase	transition to next disease phase
*between-host level*	host	transmission, death

### Waiting times

In general, whenever a new event *E* is created during the simulation at time *t*, the exact moment when *E* will take place is unknown. Therefore, we assign to *E* a threshold 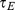 and a load 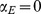. The threshold 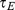 is sampled from some probability distribution 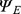 with non-negative support and mean 1. We first compute the waiting time 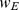, while conditioning on *E* being the first event to take place: 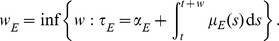



Here, 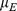 is the ‘rate’ or ‘hazard’ at which *E* takes place, which can depend on time. Notice that the (conditional) waiting time 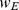 could be infinite (e.g., when the number of susceptibles equals zero, the first event to take place can never be a transmission).

When we perform this computation for all future events *E*, we find the event *F* that must take place first, and also the time at which it takes place, i.e., 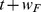. We then perform the following steps: First, we update the time 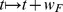. Then, for all future events *E*, we update the load 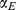 as follows: 
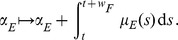



Finally, we let the event *F* act on the current state and remove *F* from our event list. For instance, if *F* happens to be a transmission event, we should initiate a new host and decrease the number of susceptible individuals. Additionally, new transmission events should be created for the transmitter and recipient. Hereafter, we re-compute the waiting times 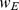 for all events *E* and repeat the above steps.

In most cases, the computation of *w*_E_ and the updating of *α*_E_ is simple. For instance, if *E* is a reversion event and *f*>0, then 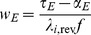. For updating the load, we replace *α*_E_ with 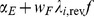. A transmission event requires more effort, because the rate of transmission varies during an individual's infectious lifetime.

The model can now be described by specifying for the events *E* listed in Table 2, their threshold-distribution 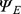, their ‘rates’ 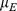, and the precise actions on the state (see Table 3).

**Table 3:** Threshold distributions, rates, and actions for the events in the model.

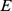	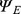	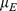	actions
*within-host level*			
escape mutation	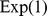	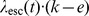	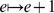 , create new escape mutation event
reversion	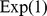	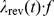	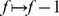 , create new reversion event
phase change ( 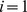 )	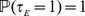	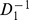	change the phase into ‘asymptomatic’, create a new phase change event
phase change ( 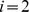 )	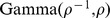	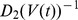	change the phase into ‘AIDS phase’, create a new phase change event
phase change ( 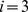 )	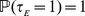	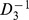	end of the within-host simulation
*between-host level*			
transmission	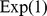	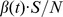	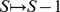 , create a new infected individual, create a new transmission event for both the transmitting and the receiving host
death	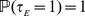	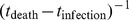	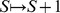 , remove the deceased host

The functions 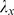 for 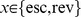 are here defined by 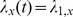, if the patients disease is in the acute phase, and similarly 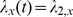 for the asymptomatic phase, and 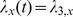 for the AIDS phase. The function 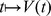 describes the viral load during the asymptomatic phase (that may not be constant due to escape mutations and reversions). The shape parameter 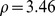 for the Gamma distribution was estimated by Fraser et al. [17].

### Stochastic computation of the next-generation matrix

In order to study the steady state of the above described model, we developed a faster and more accurate method. In deterministic (e.g. ODE-based) models with multiple viral strains, one can compute the next-generation matrix (NGM), using the model's rate equations [67]. Given a ‘generation’ (i.e., a distribution of strains in a cohort of newly infected individuals), the NGM gives the ‘next generation’ after mutation and selection in a discrete generation-based model. The steady states of the original (continuous time) and generation-based model coincide. This steady state can be computed by finding the dominant eigenvector of the NGM. The dominant eigenvalue equals (by definition) 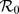 [68].

Our model is not deterministic, but we can approximate the NGM using a Monte-Carlo method. We start with a virus that has *m*_1_ mutations. We then infect a large cohort (of size *N*) of individuals. These individuals may have different binding repertoires (of diverse size *k*), so we first sample pairs (*e*_1_,*f*_1_) with 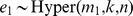 and 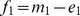. Then we run a within-host simulation for each of the virus-host pairs. Finally, we sample strains (*e*,*f*) that would be transmitted by the hosts at the start of an epidemic, and we count the number of transmitted stains 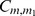 that have 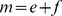 mutations. The vector 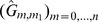 with 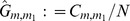 approximates the *m*_1_-th column of the NGM.

If the sample size *N* is large enough, the dominant eigenvalue and corresponding right eigenvector of the matrix 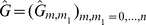 approximate, respectively, 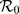 and the steady state distribution of prevalent viral strains in our agent-based model. By sampling strains from the steady-state distribution, and simulating infections with these strains, we can compute statistics as 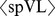 in equilibrium. This method is not based on formal arguments, but below we put forward some heuristic evidence for its correctness.

### Estimating heritability

For the statistic heritability (*h* ^2^), the above scheme is insufficient. However, we do have a cohort of potential transmitters, and hence we can create transmission couples by first sampling transmitted strains from the cohorts’ individuals, and then infecting recipients. The statistic *h* ^2^ is computed as the slope of the regression between the spVL of transmitters and receivers.

Classically, heritability of a trait *x* is defined as the proportion of variance in *x* that is caused by inherited genetic factors [see e.g. 18]. Hence, if we write 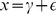, where 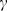 is a genetic, and 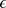 an environmental factor, then 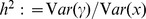. The slope of the regression mentioned above is an estimator for this quantity, but only when the transmitted quantity 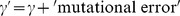 in the recipient is independent of the the transmitter's environmental factor 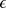. Below we will see that such an independence assumption does not hold for our model, and that the use of transmission couples results in an over-estimate of spVL heritability.

### Quantification of the footprint effect on heritability of spVL

To quantify the effect of the immunological footprint on heritability, we use a structural equation model (SEM), depicted as a directed, acyclic graph (DAG) in Figure 7. In our model, the actual inherited quantity is the number of mutated peptides 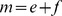. During an infection this quantity can change due to escapes and reversions, so we will only incorporate the number of mutations at the moment of infection (*m*_tra_ for a transmitting host, and *m*_rec_ for the corresponding receiver) in our statistical model.

The set-point virus load of the receiver (spVL_rec_) depends on *m*_rec_, and the breadth of the immune response against the wild-type virus (*k*_rec_). Of course, more factors determine the set-point virus load, such as the initial number of escape mutations, and stochastic effects such as mutations and progression to AIDS, but the simplified SEM only contains the variables spVL, *m* and *k*.

Apart from *k*_tra_, the breadth of the transmitter's immune response and *m*_tra_, the set-point virus load of the transmitter (spVL_tra_) depends also on *m*_rec_. This is because the set-point is an average over the chronic phase, and hence, the transmitted virus co-determines the set-point of the transmitter. In Figure 7, this is indicated by the arrow 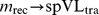.

During infection of the transmitter, the virus escapes a number of immune responses, and this number is dependent on *k*_tra_. This means that *k*_tra_ influences the number of mutations of the transmitted virus *m*_rec_. This ‘immunological footprint’ is represented by the arrow 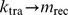 in Figure 7. The breadth of the immune response *k*_tra_ has no direct effect on *m*_tra_, since *m*_tra_ corresponds to the transmitter's founder virus. Likewise, there is no direct effect of *k*_rec_ on *m*_rec_.

We use the the R package lavaan [69] to fit the model to (standardized) simulated data, that were produced using the NGM method and the standard model's parameters. As an example, the result of one of such fits is given in Figure 7. In this graph, the numbers above the arrows indicate the estimated weights. The maximal virus load *V*_max_ equals 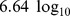 copies per ml, and the mutation rate 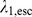 equals 3y^−1^, such that the mean set-point for this population is 4.51 log_10_ml^−1^ (cf. Figure 4A). Despite the large sample size of 25690 transmission couples, and the fact that the SEM has 4 degrees of freedom, the model describes the data quite well (the 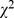-test's *p*-value equals 0.81, and the root mean square error of approximation (RMSEA) equals 0 with a 90% CI of [0,0.01]).

In the context of our SEM, the statistic *h* ^2^ equals the correlation between spVL_rec_ and spVL_tra_. This correlation can be computed as the sum of the contributions of all paths that connect spVL_tra_ with spVL_rec_. The contribution of each path equals the product of the coefficients along the path. The 3 paths that connect spVL_tra_ with spVL_rec_ are: 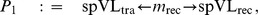


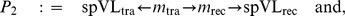


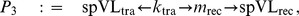
where *P*_3_ is responsible for the immunological footprint. In the example of Figure 7, the contribution of *P*_3_ equals 0.082, which is about half (49.7%) of the total correlation between spVL_rec_ and spVL_tra_ (i.e., of the heritability). We refer to the contribution of the path *P*_3_ as the “contribution of the immunological footprint to heritability”.

### A comparison of HIV-1 clades B and C

We downloaded representative sequences for clades B and C from LANL's HIV sequence database (www.hiv.lanl.gov; four sequences for each clade, as described in [70]). Then, we downloaded the HLA-A and HLA-B distributions for Europe and Sub-Saharan Africa from the NCBI database dbMHC (www.ncbi.nlm.nih.gov/projects/gv/mhc, [71]). Using the MHC binding predictor NetMHCpan (version 2.4 [45]), we computed binding affinities of all 9-mers from the representative strains for the most common HLA alleles (covering 95% of the populations). For each HLA molecule, the binding threshold was chosen such that the top 1% of a set of 10^5^ naturally occurring peptides would be considered a binder (as described in [72]).

For our analysis, we sample pairs of HLA haplotypes from the HLA distributions of one of the populations (ignoring linkage disequilibrium), each haplotype consisting of two HLA-A alleles and two HLA-B alleles. For each two haplotypes, we then compare the similarity of the binding repertoires with respect to one of the four representative strains. As a measure of similarity, we use the Jaccard index (*J*): the size of the intersection, divided by the size of the union of the two binding repertoires. This gives us the distribution of similarity scores of a population with respect to a strain. Figure 8B depicts two of these distributions. The black bars correspond to the European population with respect to a clade B virus, and the gray bars to the Sub-Saharan population with respect to a clade C virus.

By comparing the similarity distributions of a Sub-Saharan with a European population (Figure 8B), we can assess the difference in heterogeneity between the two populations and clades. The right panel of Figure 8C depicts the medians (one value for each representative strain). The European medians are significantly higher than the Sub-Saharan medians. For a better comparison between two distributions, we use a *U*-statistic, defined as 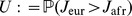, where *J*_eur_ and *J*_afr_ are distributed as the European and Sub-Saharan similarity distributions, respectively (cf. the Mann-Whitney *U*-test). Hence, *U* equals the likelihood that a random haplotype pair in the European population shows more similarity than a random pair in the Sub-Saharan population. We have four clade B strains and four clade C strains, and hence we can compute 16 probabilities *U* (Figure 8C, right panel). They turn out to be significantly higher than 0.5, meaning that the European population, subject to clade *B* strains, is less heterogeneous than the Sub-Saharan population and clade C strains.

### Deterministic computation of the NGM

We model within-host escape and reversion with two Markov chains: 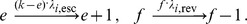



Let 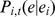 and 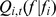 denote the probability at time *t* that during infection phase *i* the host is infected by a virus with *e* escape mutations and *f* deleterious mutations, respectively, given that phase *i* started with an 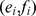-virus at time 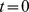. These probabilities satisfy the Kolmogorov forward equations [see e.g. 73]. 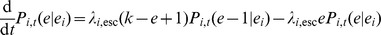


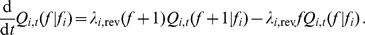



Closed-form expressions for 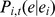 and 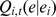 are given by 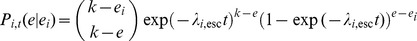


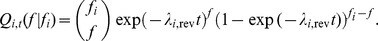



The probability 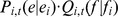 that the host is infected with an (*e*,*f*) -virus only makes sense if we condition on the infection still being in phase *i*. We want to get the expected number of transmitted virus of a specific type, and in order to make the calculations possible, we take exponential distributions for the length of the phases. We tested that this assumption is not crucial by considering Erlang distributions. The rate at which phase *i* ends is given by 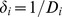. We also assume that mutation during the asymptomatic phase is slow and that the spVL is determined by the virus at the end of the acute phase (which is of type 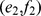). This means that 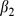 and 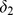 can be kept constant. Furthermore, the fraction of susceptibles (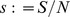) can be kept constant, either because the population is in a steady state, or because the epidemic has just started (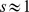).

Consider the probability generating function [cf. 74] for the number of transmitted virus of type 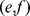 during phase 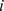: 
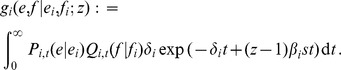



Assuming that 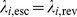, we can write this integral in terms of the Beta function (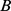). First we substitute the above given expressions for 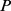 and 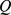


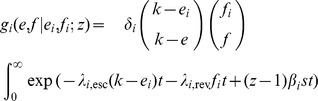


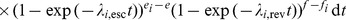
and when we now assume that 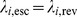, we can get



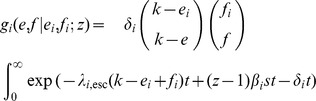


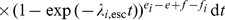


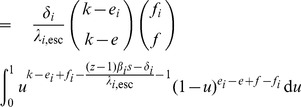
which equals by definition 
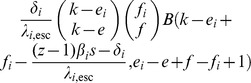
.

Since one of the arguments in this Beta function is an integer, the function 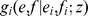 is rational: 
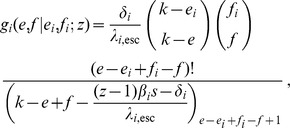
where we use the (rising) Pochhammer symbol 
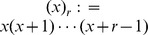
.

Now that we have this expression for 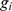, we can exploit the probability generating function's useful properties. The number 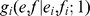 equals the probability that at the end of phase 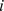, the host is infected with an 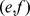-virus. The expected number of transmitted 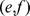-strains during phase 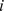 equals 
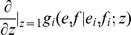
. We use the following notation: 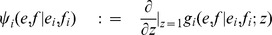


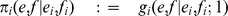



If we now take into account that a transmitted 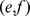-virus has a different phenotype 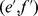 in the receiver (with probability given by the hypergeometric distribution), we can find the NGM for the case 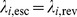. We verified that for this part of the parameter space (i.e., 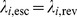), the deterministic and stochastic computation give the same results (not shown).

### The bifurcation in the model with a homogeneous host population

When the host population is homogeneous (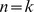), we find a threshold in the parameter space across which between-host adaptation is no longer possible. Here we will make this precise and show that this threshold is caused by a transcritical bifurcation. In a homogeneous population, we lose deleterious mutations. In the above introduced notation, we may ignore 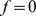 and we write for instance 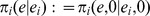. Let 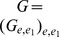 denote the NGM, then we get the following formula in terms of 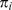 and 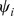: 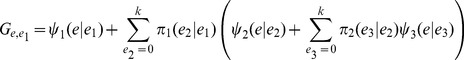



The matrix 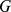 is triangular, since the number of escape mutations, which equals the total number of mutations, can only grow during an infection. The diagonal elements of 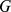 are the eigenvalues of 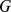, and the dominant eigenvalue equals (by definition) 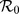 of the quasi-species. The diagonal elements can be written as 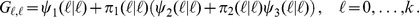



If 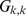 is dominant, then population-level evolution will result in strains that have escaped all CTL responses. If another eigenvalue 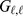 with 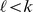 is dominant, then not all viruses have escaped all CTL responses and this is due to selection for transmission on the population-level.

If we now fix 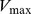 and let 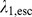 approach 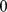 from the right, then for high 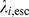 the eigenvalue 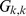 is dominant. The mentioned bifurcation occurs when 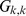 equals one of the 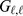 (with 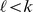) for the first time. We first give simple expressions for 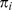 and 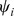 that occur in the expression for 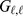: 
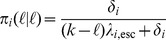


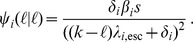



These expressions and the formula for 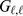 enable us to (numerically) find the curves 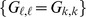 for 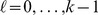. These curves and the resulting threshold are shown in Figure 6.

## Supporting Information

S1 File **The source code for the simulations.** Information about compiling the code and running the simulation can be found in the **README** file.
(GZ) Click here for additional data file.
