# Supplementary figures and images for: Immuno-epidemiological Modeling of HIV-1 Predicts High Heritability of the Set-Point Virus Load, while Selection for CTL Escape Dominates Virulence Evolution
Source: PLoS Comput Biol. 2014 Dec 18;10(12):e1003899. doi: 10.1371/journal.pcbi.1003899 (PMC4270429; doi:10.1371/journal.pcbi.1003899)

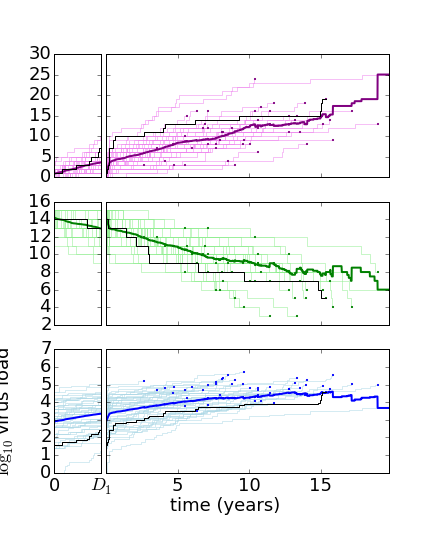

Supplement: S1 File — The source code for the simulations. Information about compiling the code and running the simulation can be found in the README file. (GZ) [file pcbi.1003899.s001.gz › hiv-model/examples/singlehost-plot.png]

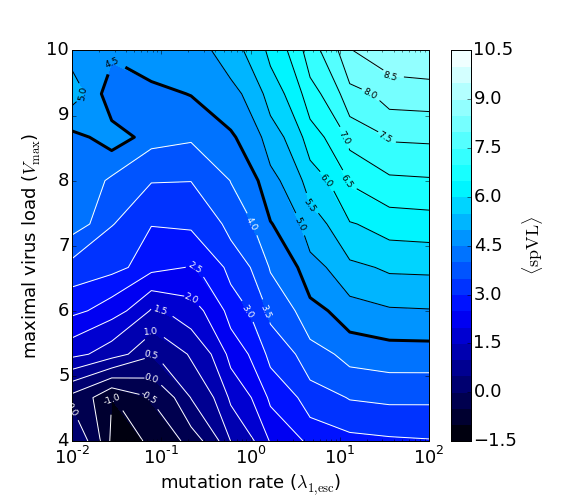

Supplement: S1 File — The source code for the simulations. Information about compiling the code and running the simulation can be found in the README file. (GZ) [file pcbi.1003899.s001.gz › hiv-model/examples/parametersweep-plot.png]

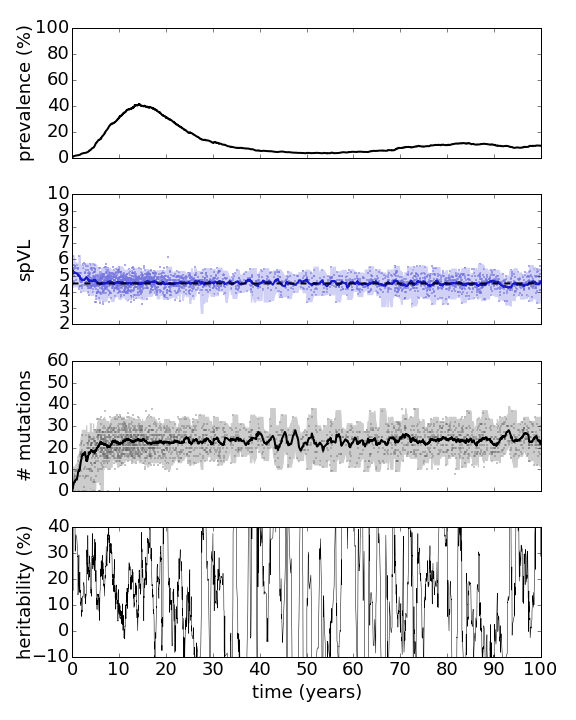

Supplement: S1 File — The source code for the simulations. Information about compiling the code and running the simulation can be found in the README file. (GZ) [file pcbi.1003899.s001.gz › hiv-model/examples/epidemic-plot.png]
